# Physical activity and breast cancer risk: results from the UK Biobank prospective cohort

**DOI:** 10.1038/s41416-019-0700-6

**Published:** 2020-01-10

**Authors:** Wenji Guo, Georgina K. Fensom, Gillian K. Reeves, Timothy J. Key

**Affiliations:** Cancer Epidemiology Unit, Nuffield Department of Population Health, University of Oxford, Oxford, UK

**Keywords:** Risk factors, Epidemiology, Breast cancer

## Abstract

**Background:**

Previous studies suggest a protective role of physical activity in breast cancer risk, largely based on self-reported activity. We aimed to clarify this association by examining breast cancer risk in relation to self-reported physical activity, informed by accelerometer-based measures in a large subset of participants.

**Methods:**

We analysed data from 47,456 premenopausal and 126,704 postmenopausal women in UK Biobank followed from 2006 to 2014. Physical activity was self-reported at baseline, and at resurvey in a subsample of 6443 participants. Accelerometer data, measured from 2013 to 2015, were available in 20,785 women. Relative risks (RRs) and 95% confidence intervals (CIs) were calculated by using multivariable-adjusted Cox regression.

**Results:**

A total of 3189 cases were diagnosed during follow-up (mean = 5.7 years). Women in the top compared with the bottom quartile of self-reported physical activity had a reduced risk of both premenopausal (RR 0.75; 95% CI 0.60–0.93) and postmenopausal breast cancer (RR 0.87; 95% CI 0.78–0.98), after adjusting for adiposity. In analyses utilising physical activity values assigned from accelerometer measurements, an increase of 5 milli-gravity was associated with a 21% (RR 0.79; 95% CI 0.66–0.95) reduction in premenopausal and a 16% (RR 0.84; 95% CI 0.73–0.96) reduction in postmenopausal breast cancer risk.

**Conclusions:**

Greater physical activity is associated with a reduction in breast cancer risk, which appears to be independent of any association it may have on risk through its effects on adiposity.

## Introduction

Previous prospective studies have assessed the association between self-reported physical activity and breast cancer, with overall findings that support a protective role for physical activity in breast cancer risk.^1–3^ However, self-reported responses from questionnaires are prone to both inaccurate reporting and bias, leading to random and systematic error, respectively.^4^ Further research incorporating objective methods of physical activity assessment, such as accelerometers, is needed to better understand this measurement error and reduce misclassification of physical activity levels.

Some studies have also reported greater reductions in risk associated with physical activity among postmenopausal women^1^ and those with a relatively low body mass index (BMI),^5^ but it is unclear as to what extent these observed differences may be attributed to differential reporting bias in self-reported estimates of physical activity by subgroups of menopausal status and BMI, as opposed to genuine effect modification.

The purpose of this study was to assess the associations between physical activity and breast cancer risk in over 47,000 premenopausal and 126,000 postmenopausal women, by using baseline self-reported physical activity in conjunction with repeat questionnaires and subsequently measured accelerometer values in large subsamples to better inform the extent to which self-reported estimates may be biased and to reduce misclassification of physical activity level. We also examined whether and to what extent the relationship between physical activity and breast cancer risk may be mediated by adiposity using an objective measure of body fat assessed by bioelectrical impedance, previously shown to be strongly associated with breast cancer risk.^6^ This research has been conducted using the UK Biobank Resource, a nationwide study of ~500,000 UK adults.

## Methods

### Data source

Data were obtained from UK Biobank (reference number 3248, approved August 2013). Details of the rationale, design and survey methods for UK Biobank have been described fully elsewhere,^7^ and information on data availability and access procedures is on the study website (http://www.ukbiobank.ac.uk/).

### Study participants

The complete UK Biobank dataset includes 502,620 UK adults (229,165 men and 273,455 women) aged between 40 and 70 years at recruitment during 2006–2010. Postal invitations were mailed to individuals registered with the National Health Service who lived within ~25 miles of a UK Biobank assessment centre. The response rate in women was 6.4%.^8^ As described previously,^6^ participants completed a self-administered touchscreen questionnaire during the baseline assessment centre visit that included questions on sociodemographics, lifestyle, health and medical history and sex-specific factors. Physical measurements on the whole cohort, including body size and composition, were also assessed during the baseline assessment centre visit. Between August 2012 and June 2013, a repeat assessment of all baseline measures, including physical activity, was conducted in 20,345 participants at the UK Biobank Coordinating Centre in Stockport.

Women were excluded from the analyses if they had a prior cancer diagnosis (except for non-melanoma skin cancer ICD-10 C44) (*n* = 18,372), had missing data on any of the physical activity variables used in our analyses (*n* = 65,291), had a sum of all walking, moderate and vigorous physical activity greater than 6720 min per week (*n* = 2021), as recommended in the International Physical Activity Questionnaire (IPAQ) guidelines,^9^ or had missing data on body fat mass (*n* = 3163) (Supplementary Fig. 1).

The final analyses included 47,456 premenopausal and 126,704 postmenopausal women. Women were defined as being premenopausal or postmenopausal at recruitment based on whether they reported that their periods had stopped; for these analyses, women with unknown self-reported menopausal status who were under the age of 45 who had not undergone a bilateral oophorectomy were categorised as premenopausal and women at the age of 53 or over and/or had both ovaries removed were categorised as postmenopausal, based on previously established criteria.^10^ Women who had unknown menopausal status were excluded from the subgroup analysis by menopausal status (*n* = 10,448) (Supplementary Fig. 1).

As described previously,^11^ physical activity questions from the baseline questionnaire captured the frequency and duration of three levels of activity (walking, moderate and vigorous). Participants were asked how many days per week they typically engaged in each category of activity. For each category in which an answer of one or more days was given, the participant was subsequently asked the number of minutes typically spent on the activity per day. Questions were adapted from the IPAQ, a validated survey instrument,^9^ and are available to view on the UK Biobank website (http://biobank.ctsu.ox.ac.uk/crystal/). Metabolic equivalents (METs) were used to quantify self-reported physical activity; 1 MET is expended by sitting quietly for 1 h, and the MET value reflects the ratio of energy expended per kilogram of body weight per hour to that expended when sitting quietly.^12^ The number of minutes per day engaged in each level of activity was multiplied by the respective MET score for the corresponding level of activity. MET minutes per day were converted to MET hours per week. The total amount of METs was calculated by summing the total METs from the walking, moderate and vigorous activity levels. Following IPAQ guidelines,^9^ physical activity of less than 10 min per day for any category was recoded to 0. The following MET values were used, as recommended by IPAQ: 3.3 for walking, 4.0 for moderate physical activity and 8.0 for vigorous physical activity.^9^

Between February 2013 and December 2015, 236,519 participants, all of whom had provided a valid email address, were invited to participate in a 7-day accelerometer study (on average, ~5.5 years after recruitment). Beginning in June 2013, participants were sent wrist-worn triaxial accelerometers (Axivity AX3, Newcastle upon Tyne, UK) that were programmed to capture three-dimensional acceleration data at 100 Hz with a dynamic range of ±8 g. Participants were provided with instructions to wear the accelerometer on their dominant wrist continuously for 7 days and then to return the device to the coordinating centre using the prepaid envelope provided. Researchers affiliated with UK Biobank extracted physical activity information from raw 100-Hz triaxial acceleration data after calibrating the data, removing gravity and sensor noise and identifying wear and non-wear episodes. In the present analyses, the “overall acceleration average” variable was used (data field 90012), which is the average vector magnitude of acceleration. Further details on data collection and processing can be found elsewhere.^13^

### Anthropometry and body composition

At the baseline interview, height and weight were measured, and the Tanita BC-418MA body composition analyser was used to measure body fat mass using bioimpedance. BMI was calculated by dividing weight (kg) by the square of standing height (m^2^).

### Ascertainment of cancer cases

Data on cancer diagnoses and deaths were obtained by UK Biobank through National Health Service (NHS) Digital for participants in England and Wales and NHS Central Register for participants in Scotland. Completeness of case ascertainment in English cancer registries is reported to be approximately 98–99%, based on a study that linked routine cancer registration with information from the Hospital Episode Statistics database.^14^

### Statistical analyses

Baseline characteristics of participants were summarised by self-reported physical activity separately for pre- and postmenopausal women. As described previously,^6^ women were followed from the date of baseline assessment centre visit until the earliest of the date of breast cancer registration (ICD-10 C50), date of death, date of loss to follow-up or end of follow-up for cancer incidence (30 November 2014). Women diagnosed with any cancer other than breast cancer (with the exception of non-melanoma skin cancer) during follow-up were censored at the date of diagnosis.

Multivariable-adjusted Cox regression with attained age as the underlying time variable was used to estimate hazard ratios (referred to as relative risks [RRs]) and 95% confidence intervals (CIs) for the associations between self-reported physical activity and breast cancer risk. In the tables, RRs are presented with group-specific confidence intervals (95% g-s CI) for the log risk in each group, which allows comparisons to be made between any two categories, even if neither is the reference group.^15^ Conventional 95% CIs are reported in the text.

For the risk analyses, baseline self-reported physical activity was categorised into quartiles based on the distribution in all women. Linear trends were calculated by assigning the median value of repeat self-reported physical activity within each category of baseline physical activity. In additional analyses incorporating an objective measure of physical activity, tests for linear trend were performed with categories assigned the median of the accelerometer-based physical activity measurement within each baseline self-reported physical activity category;^16^ accelerometer data were not used from participants with poor wear time (*n* = 2815) or poor data calibration (*n* = 4) due to insufficient data, or those in whom accelerometer data were collected after breast cancer diagnosis or the end of follow-up (*n* = 22,467). Linear trends are reported per 5 milli-gravity of accelerometer-based physical activity score. Assessment of interaction terms between each exposure of interest and the underlying time variable did not suggest significant deviation from proportional hazards.

Variables that were associated with both the exposure of interest and causally associated with the outcome were included in regression models as possible confounders. Covariates were also selected a priori based on potential risk factors for breast cancer. All analyses were stratified by 5-year age at recruitment categories, region of recruitment and socioeconomic status (based on quintiles of Townsend deprivation index^17^), and adjusted for family history of breast cancer (no, yes), height (<160, 160–165.9 and ≥166 cm), number of births (nulliparous, 1–2, ≥3 births), age at first birth (<25, 25–29 and ≥30 years), age at menarche (<12, 12–13 and ≥14 years), oral contraceptive use (never, previous and current), ever had breast cancer screening (no, yes), smoking status (never, previous and current) and alcohol intake frequency (less than 3 times a month, 1–4 times a week, daily or almost daily). Analyses for postmenopausal women were additionally adjusted for hormone replacement therapy (HRT) use (never, previous and current) and age at menopause (<45, 45–49, 50–54 and ≥55 years). Analyses were further adjusted for body fat mass in multivariable-adjusted models that included height. In models that included all women, the association of body fat mass with risk was allowed to vary by menopausal status by including a variable generated by the cross-classification of body fat mass and menopausal status.

Women with missing values for any of the adjustment variables were assigned to a separate “unknown” category for the respective variable. Information was either missing or reported as unknown for <3% of each covariate, with the exception of age at menopause (19.3%) among postmenopausal women, which can be difficult to report accurately since menopausal status can be obscured by hysterectomy or HRT use before menstrual periods stop.^10^ A sensitivity analysis restricted to participants with known values for all adjustment variables was conducted to assess the impact of missing values. We also conducted sensitivity analyses to assess the impact of excluding the first 2 years of follow-up, excluding those who reported long-term illness, disability or infirmity, excluding those who reported poor health and excluding follow-up for women once they reached the age of 50 in the premenopausal analysis.

A chi-squared test for heterogeneity was used to assess whether menopausal status was an effect modifier in the association between accelerometer-based physical activity score and breast cancer risk, as well as in the association between self-reported physical activity and breast cancer risk. Chi-squared tests for heterogeneity were also used to determine whether the trend in breast cancer risk per 50 MET hours of self-reported physical activity was modified by BMI (<25 kg/m^2^, ≥25 kg/m^2^), number of births (0–1, ≥2 births), HRT use (never, ever) and alcohol intake (less or more than one drink weekly). Based on IPAQ data processing and analysis guidelines, 50 MET hours were chosen as the approximate threshold for achieving a “high” level of physical activity.^18^ All analyses were conducted using STATA version 15.0 (Stata Corp LP, College Station, TX).

## Results

A total of 3189 invasive breast cancer cases were diagnosed among 184,608 women during a mean follow-up of 5.7 (standard deviation 1.1) years. Of these, 717 cases were diagnosed among 47,456 women who were premenopausal at recruitment, and 2315 invasive breast cancer cases were diagnosed among 126,704 women who were postmenopausal at recruitment. In these analyses, accelerometer data were available on a subset of 20,785 women. We examined self-reported physical activity in relation to potential confounders for breast cancer (Table 1). Premenopausal women who did the least physical activity had a BMI of 27.5 kg/m^2^ compared with 25.6 kg/m^2^ in those who did the most physical activity on average. Postmenopausal women who did the least physical activity had a BMI of 28.2 kg/m^2^ compared with 26.3 kg/m^2^ in those who did the most physical activity on average. A slightly greater proportion of women in the top quartile of physical activity consumed alcohol on a weekly or more frequent basis. Compared with premenopausal women, postmenopausal women reported more physical activity but were objectively less physically active, as measured by the accelerometer. The most active postmenopausal women reported an average of 94.9 MET hours/week compared with 91.8 MET hours/week of physical activity in the most active premenopausal women. The objectively measured physical activity value for the most active postmenopausal women was 30.3 milli-gravity compared with 34.4 milli-gravity in the most active premenopausal women.

**Table 1:** Characteristics of the study population at recruitment, according to self-reported physical activity and menopausal status.

	Premenopausal		Postmenopausal	
Quartile of physical activity	Bottom quartile (least active)	Top quartile (most active)	Bottom quartile (least active)	Top quartile (most active)
Age at recruitment (years), mean (SD)	45.7 (3.6)	45.7 (3.5)	59.3 (5.5)	60.4 (5.5)
Lowest socioeconomic quintile, %	22.4	24.3	19.5	19.0
Family history of breast cancer, %	5.7	5.7	6.6	6.6
Age at menarche (years), mean (SD)	13.0 (1.6)	13.1 (1.7)	12.9 (1.6)	13.0 (1.6)
Age at first birth (years), mean (SD)	28.2 (5.6)	27.3 (5.3)	25.7 (5.0)	25.2 (4.8)
Parity, mean (SD)	1.5 (1.2)	1.7 (1.2)	1.8 (1.2)	1.9 (1.2)
Ever oral contraceptive use, %	89.6	89.4	81.1	78.0
Age at menopause (years), mean (SD)			49.7 (5.0)	49.7 (5.2)
Current hormone replacement therapy use, %			49.8	51.5
Current smokers, %	11.0	11.0	9.3	7.7
One or more drinks weekly, %	63.3	64.5	61.2	63.0
Height (cm), mean (SD)	164.1 (6.3)	164.2 (6.2)	162.1 (6.3)	161.9 (6.1)
Body mass index (kg/m^2^), mean (SD)	27.5 (5.9)	25.6 (4.7)	28.2 (5.6)	26.3 (4.5)
Body fat mass (kg), mean (SD)	27.9 (11.6)	23.5 (9.4)	29.5 (10.8)	25.3 (8.8)
Plumper than average at age 10, %	20.3	18.2	19.1	16.0
Total physical activity (MET hrs/week), median (IQR)	7.1 (3.8, 10.4)	91.8 (71.3, 134.1)	7.4 (4.0, 10.6)	94.9 (72.4, 136.5)
Accelerometer physical activity (milli-gravity), mean (SD)*	28.3 (7.4)	34.4 (9.7)	25.7 (6.7)	30.3 (8.2)

*MET hrs/week* metabolic equivalent hours per week; *SD* standard deviation

*Data were collected after recruitment between February 2013 and December 2015

Self-reported physical activity was associated with breast cancer risk reduction in both premenopausal and postmenopausal women (Table 2). Premenopausal women in the top quartile of physical activity, who reported 58.3 or more MET hours/week of physical activity, had a 23% decreased risk of breast cancer (RR 0.77; 95% CI 0.62–0.96) compared with those in the bottom quartile. The protective association was marginally strengthened after further adjusting for body fat mass (RR 0.75; 95% CI 0.60–0.93). Among postmenopausal women, the top quartile of physical activity was associated with a 17% decreased risk of breast cancer (RR 0.83; 95% CI 0.74–0.93) compared with the bottom quartile. Further adjustment for body fat mass attenuated this protective association (RR 0.87; 95% CI 0.78–0.98). The results for linear trend per 50 MET hours/week of self-reported physical activity were RR 0.74 (95% CI 0.60–0.92) for premenopausal women and RR 0.85 (95% CI 0.76–0.96) for postmenopausal women (fully adjusted models), with no heterogeneity by menopausal status (*p* = 0.26) (Table 2).

**Table 2:** Association between physical activity and risk of invasive breast cancer by menopausal status in UK Biobank (*n* = 174,160 women followed from 2006 to 2014).

Self-reported physical activity quartiles	Median self-reported physical activity	Repeat median self-reported physical activity	Median accelerometer physical activity	Cases/total (*n*)	Relative risk	Relative risk
(MET hrs/week)	(MET hrs/week)	(MET hrs/week)	(milli-gravity/week)		(95% g-s CI)^a^	(95% g-s CI)^b^
*Premenopausal women*						
0–13.78	7.30	15.40	27.34	205/12,324	1.00 (0.87–1.15)	1.00 (0.87–1.15)
13.79–29.55	21.12	23.10	29.47	200/12,318	0.97 (0.84–1.11)	0.95 (0.83–1.09)
29.56–58.27	40.80	34.22	31.25	183/12,261	0.91 (0.79–1.05)	0.89 (0.77–1.03)
≥58.28	91.80	64.40	33.24	129/10,553	0.77 (0.65–0.92)	0.75 (0.63–0.89)
Linear trend per 50 MET hours/week (corrected with repeat self-reported physical activity)					0.77 (0.62–0.95)	0.74 (0.60–0.92)
P for trend					0.015	0.008
Linear trend per 5 milli-gravity (using median accelerometer values)					0.82 (0.69-0.97)	0.79 (0.66-0.95)
P for trend					0.022	0.010
*Postmenopausal women*						
0–13.78	7.53	13.20	25.12	614/31271	1.00 (0.92–1.08)	1.00 (0.92–1.08)
13.79–29.55	21.17	23.89	26.83	611/31,196	0.99 (0.91–1.07)	1.01 (0.94–1.10)
29.56–58.27	41.10	38.75	27.77	553/31255	0.88 (0.81–0.96)	0.92 (0.84–1.00)
≥58.28	95.10	63.10	29.39	537/32,982	0.83 (0.76–0.90)	0.87 (0.80–0.95)
Linear trend per 50 MET hours/week (corrected with repeat self-reported physical activity)					0.81 (0.73–0.91)	0.85 (0.76–0.96)
P for trend					0.000	0.006
Linear trend per 5 milli-gravity (using median accelerometer values)					0.79 (0.69–0.90)	0.84 (0.73–0.96)
P for trend					<0.001	0.010
P for heterogeneity by menopausal status (corrected with repeat self-reported physical activity) = 0.26						
P for heterogeneity by menopausal status (using median accelerometer values) = 0.61						

*MET hrs/week* metabolic equivalent hours per week; *g-s CI* group-specific confidence interval

^a^Stratified by age at recruitment, region of recruitment and socioeconomic status. Adjusted for family history of breast cancer, height, parity, age at menarche, oral contraceptive use, age at first birth, smoking status, ever had breast cancer screening, alcohol intake frequency, age at menopause and HRT use (only in postmenopausal women)

^b^Further adjusted for quartiles of body fat mass within each menopausal status group

For linear trends according to accelerometer-based physical activity, an increase of 5 milli-gravity was associated with a 18% reduction in premenopausal breast cancer risk before adjustment for body fat mass (RR 0.82; 95% CI 0.69–0.97) and a 21% reduction after adjustment for body fat mass (RR 0.79; 95% CI 0.66–0.95). The corresponding relative risks for postmenopausal breast cancer were 0.79 (95% CI 0.69–0.90) before adjustment for body fatness and 0.84 (95% CI 0.73–0.96) after adjustment for body fatness. The fully adjusted trends in risk per additional 5 units of milli-gravity did not differ significantly by menopausal status (*p* = 0.61) (Table 2).

**Fig. 1:**
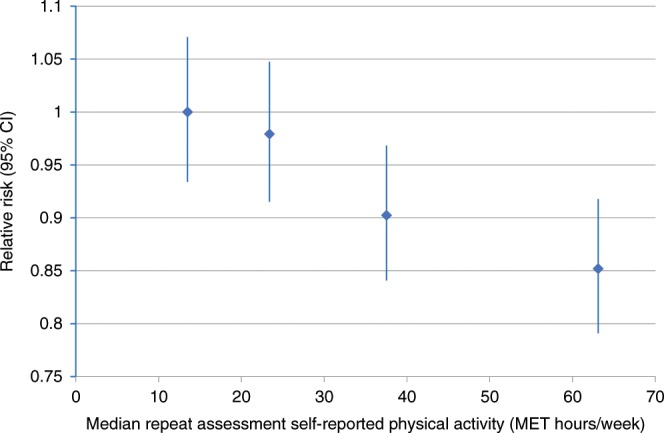
Association between physical activity and risk of invasive breast cancer. Self-reported physical activity was grouped into quartiles. Relative risk for the association between self-reported physical activity and risk of invasive breast cancer is plotted against the median repeat assessment physical activity value within each quartile of baseline self-reported physical activity. The analysis is stratified by age at recruitment, region of recruitment and socioeconomic status, and is adjusted for family history of breast cancer, height, number of births, age at menarche, age at first birth, oral contraceptive use, ever had breast cancer screening, smoking status, alcohol intake frequency, hormone replacement therapy use, age at menopause and a variable generated by the cross-classification of body fat mass and menopausal status. The figure shows point estimates and 95% group-specific confidence intervals. CI, confidence interval.

Figure 1 illustrates the association between self-reported physical activity and breast cancer risk for all women combined. Compared with women in the bottom quartile of self-reported activity, those in the top quartile had a 15% reduced risk of breast cancer (RR 0.85; 95% CI 0.77–0.94).

**Fig. 2:**
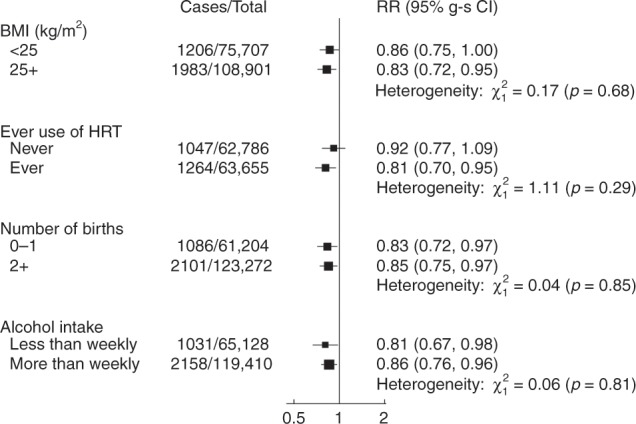
Relative risk of physical activity and breast cancer risk by BMI, ever use of HRT, number of births and alcohol intake. Analyses are stratified by age at recruitment, region of recruitment and socioeconomic status, and are adjusted for family history of breast cancer, height, number of births, age at menarche, age at first birth, oral contraceptive use, ever had breast cancer screening, smoking status, alcohol intake frequency, hormone replacement therapy use, age at menopause and a variable generated by the cross-classification of body fat mass and menopausal status. Analyses of BMI <25 kg/m^2^ and 25 kg/m^2^ or more were adjusted for a BMI group-specific variable generated by the cross-classification of body fat mass and menopausal status instead of the overall cross-classified variable. Analyses stratified by ever use of hormone replacement therapy were restricted to postmenopausal women. The figure shows the linear trend per 50 MET hours/week of self-reported physical activity for each subgroup and 95% group-specific confidence intervals. BMI, body mass index; HRT, hormone replacement therapy; RR, relative risk. Note: The number of cases does not sum to the total, as information on ever use of HRT was missing for 4 postmenopausal cases and information on the number of births was missing for 2 cases.

When trends in breast cancer risk per 50 additional MET hours of self-reported physical activity were estimated separately in subgroups of women defined by BMI, HRT use, parity and alcohol intake, there was no evidence of any significant heterogeneity in the magnitude of association according to any of these factors (Fig. 2).

## Discussion

In this large prospective study, women in the top versus bottom quartile of self-reported physical activity had an approximately 23% reduced risk of breast cancer in premenopausal women and 17% reduced risk in postmenopausal women; the data from accelerometers in a subset of women confirmed that the categories of self-reported activity data are reliably ranked according to this objective measure. Adjusting for body fat mass slightly strengthened the inverse association in premenopausal women but modestly attenuated the association in postmenopausal women, and the adiposity-adjusted trends in risk with increasing physical activity score did not vary significantly by menopausal status. These findings suggest a protective effect of physical activity on breast cancer risk for all women, beyond the role of adiposity. The main results are in agreement with a recent meta-analysis demonstrating a 19% reduced risk of breast cancer among premenopausal women and a 12% reduced risk among postmenopausal women in cohort studies that adjusted or matched for any measure of body fatness.^2^

In postmenopausal women, our finding that adjusting for body fat mass attenuated the protective association between physical activity and breast cancer is consistent with results from a previous postmenopausal cohort,^19^ although other cohort studies in postmenopausal women found minimal change after adjusting for self-reported BMI.^20,21^ In our analyses, adjusting for body fat mass measured using bioimpedance rather than self-reported BMI may partly explain the slightly greater magnitude of attenuation compared with that reported in prior studies.

Since high BMI is associated with increased breast cancer risk in postmenopausal women,^22^ the association of physical activity with breast cancer risk observed before adjustment for body fat mass is likely to be partly mediated by adiposity. Higher levels of body fat have been hypothesised to increase postmenopausal breast cancer risk through oestrogen-stimulated carcinogenesis^23,24^ due to higher circulating concentrations of oestradiol and lower concentrations of sex hormone-binding globulin, resulting in increased oestrogen bioavailability.^25^

In contrast to the association in postmenopausal women, high BMI is generally inversely associated with breast cancer risk in premenopausal women,^26^ and as expected, adjusting for adiposity slightly strengthened the protective association between physical activity and breast cancer risk. Therefore, the protective association between physical activity and breast cancer risk in premenopausal women is unlikely to be due to residual confounding by BMI. The mechanism underlying the association between physical activity and breast cancer risk reduction observed in both premenopausal and postmenopausal women, after adjustment for adiposity, remains unclear but may involve pathways, such as improved insulin sensitivity, reduced chronic inflammation and enhanced immune function.^27–29^

A recent meta-analysis of 18 cohort and 11 case–control studies found that self-reported physical activity was associated with the greatest magnitude reduction in breast cancer risk among women with lower BMI, and that there was no significant association between self-reported physical activity and breast cancer risk among women who were obese.^5^ One factor that may partly explain the lack of risk reduction associated with physical activity in some studies is the over-reporting of physical activity among individuals with higher BMI.^30^ In our analyses, the decreased risk associated with physical activity did not appear to vary by BMI and this lack of variation in association with BMI is consistent with that from a recent study that pooled data from 10 prospective cohorts with self-reported physical activity as the exposure and incident breast cancer as the outcome.^3^

The strengths of our study include the prospective design, large sample size, virtually complete follow-up^14^ and the availability of data on a wide range of potential confounders. A further notable strength of our study is the availability of objectively measured accelerometer-based physical activity. Another strength of our study is the availability of objectively measured body fat, because self-reported measures of adiposity, such as weight, tend to be underestimated by overweight and obese women.^31,32^ The limitations include the fact that the objectively measured physical activity data were only available in a subset of the cohort and the inherent limitations of the self-reported physical activity measures. It is also important to note that the accelerometers do not assess the same facets of physical activity as those assessed by the questionnaires, and the unit of measurement for accelerometer-measured physical activity currently available in the UK Biobank cannot be directly compared with MET hours of self-reported physical activity, although physical activity phenotypes are being developed, which will improve the interpretability of the accelerometer data in the UK Biobank.^33^ This study is also limited by the lack of available information on hormone receptor status, stage and grade of the tumour.

In conclusion, our results show a protective association between self-reported physical activity and breast cancer risk in both pre- and postmenopausal women. Even after adjusting for body fat mass, a similar magnitude of inverse association between physical activity and breast cancer risk remained in both groups and may be mediated by non-adiposity-related factors. Future research should aim to further clarify the mechanisms underlying this reduction in risk associated with physical activity.

## Supplementary information

Supplementary Figure 1 legend

Supplementary Figure 1

## Data Availability

Information about data access is available online (http://www.ukbiobank.ac.uk/wp-content/uploads/2011/11/UK-Biobank-Protocol.pdf).
