# Supplementary material for: Physical activity and breast cancer risk: results from the UK Biobank prospective cohort
Source: Br J Cancer. 2020 Jan 10;122(5):726–32. doi: 10.1038/s41416-019-0700-6 (PMC7054300; doi:10.1038/s41416-019-0700-6)
Supplement: Supplementary file 1 — Supplementary Figure 1 legend [file 41416_2019_700_MOESM1_ESM.docx]

**Supplementary Figure 1.** Flowchart illustrating the application of exclusion criteria for the current study
