# Supplementary figures and images for: Physical activity and breast cancer risk: results from the UK Biobank prospective cohort
Source: Br J Cancer. 2020 Jan 10;122(5):726–32. doi: 10.1038/s41416-019-0700-6 (PMC7054300; doi:10.1038/s41416-019-0700-6)

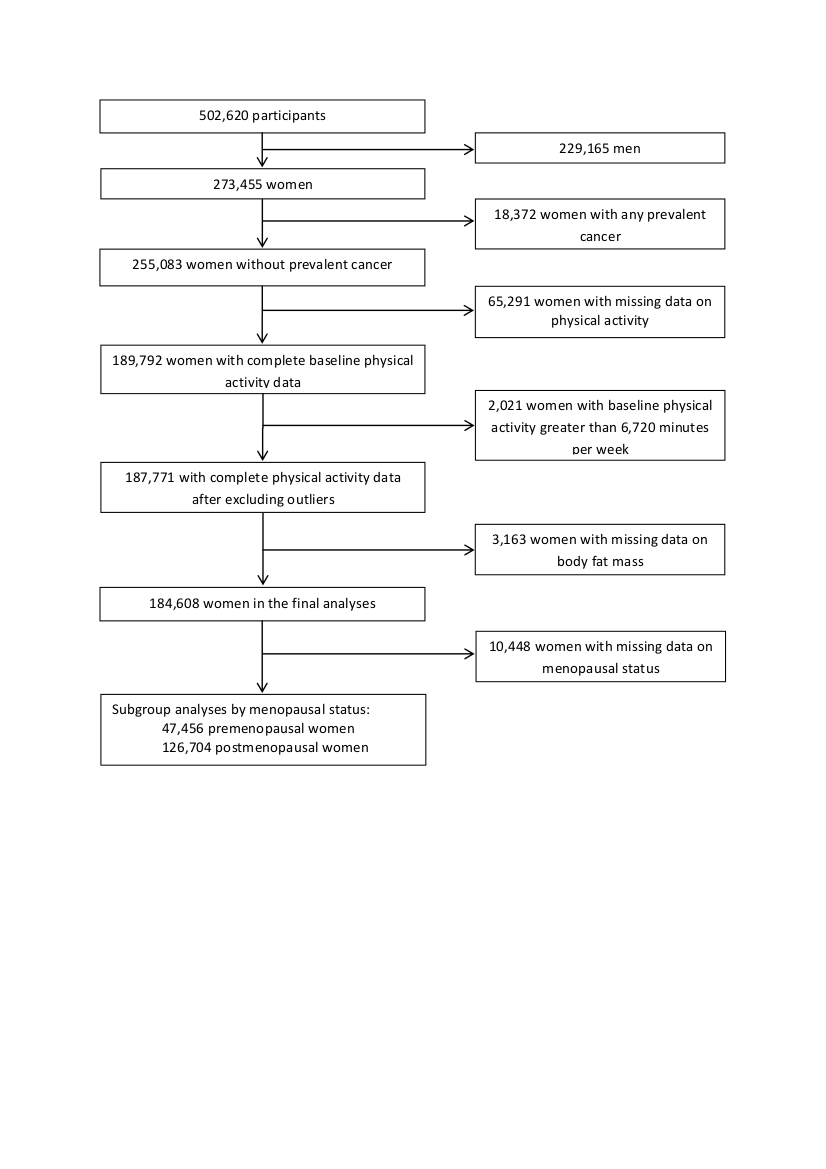

Supplement: Supplementary file 2 — Supplementary Figure 1 [file 41416_2019_700_MOESM2_ESM.gif]
